# Synergistic mechanism between the endoplasmic reticulum and mitochondria and their crosstalk with other organelles

**DOI:** 10.1038/s41420-023-01353-w

**Published:** 2023-02-09

**Authors:** Yaozhi Zhang, Yang Wu, Minjie Zhang, Zixian Li, Bin Liu, Huafeng Liu, Junfeng Hao, Xiaoyu Li

**Affiliations:** 1 Shunde Women and Children’s Hospital, Guangdong Medical University, Foshan, 528399 China; 2 Institute of Nephrology, and Guangdong Provincial Key Laboratory of Autophagy and Major Chronic Non-communicable Diseases, Affiliated Hospital of Guangdong Medical University, Zhanjiang, 524001 China

**Keywords:** Energy metabolism, Endosomes

## Abstract

Organelles are functional areas where eukaryotic cells perform processes necessary for life. Each organelle performs specific functions; however, highly coordinated crosstalk occurs between them. Disorder of organelle networks often occur in various diseases. The endoplasmic reticulum (ER) and mitochondria are crucial organelles in eukaryotic cells as they are the material synthesis and oxidative metabolism centers, respectively. Homeostasis and orchestrated interactions are essential for maintaining the normal activities of cells. However, the mode and mechanism of organelle crosstalk is still a research challenge. Furthermore, the intricate association between organelle dyshomeostasis and the progression of many human diseases remains unclear. This paper systematically summarized the latest research advances in the synergistic mechanism between the endoplasmic reticulum and mitochondria and their crosstalk with other organelles based on recent literature. It also highlights the application potential of organelle homeostasis maintenance as a preventative and treatment strategy for diseases.

## Facts

- Organelles destruction and their abnormal crosstalk impede disorder of the internal environment and drive disease progression.
- ER and mitochondria are the material synthesis center and the material oxidative metabolism center, respectively. Normal cellular metabolism is dependent on their functional activities and their unhindered crosstalk.
- Both ER and mitochondria have contact sites with other organelles, such as peroxisomes, Golgi apparatus and lysosome.

## Open questions

- How mitochondrial fission is associated with mtDNA replication?
- What are the targets and methods of organelle interaction intervention with clinical application prospects?
- When can we ascertain the complete network of organelle interactions?

## Introduction

Organelles, which are membrane structures with specific morphologies and functions, are the specialized areas in which eukaryotic cells perform activities necessary for life. The spatial regionalization and functional differentiation of organelles are being recognized with ongoing research in the fields of biochemistry and molecular biology. Cellular homeostasis is maintained by organelle cooperation and contact, which enables rapid material and information exchange and execution of various biological processes under different conditions. Organelle crosstalk network stability is highly significant in the internal environment. However, little is known about the mechanisms and functions of organelle interactions.

Increasing evidence suggests that defects in organelle communication may play a role in the pathogenesis of many human diseases. Abnormal organelle functions or structures are observed in various diseases, including cancer [1], Alzheimer’s disease [2], Parkinson’s disease [3], and amyotrophic lateral sclerosis with associated frontotemporal dementia [4]. Moreover, organelle destruction impedes their crosstalk and disorder of the internal environment drives disease progression [5].

The endoplasmic reticulum (ER) and mitochondria are the most important organelles in eukaryotic cells. The ER is the center of cell information exchange, while the mitochondria are the centers of energy metabolism [6]. Hence, these two organelles are important for maintaining cellular homeostasis. Damage to the ER and mitochondria is observed in many diseases, including tumors, neurodegenerative diseases, and diabetes [7]. Abnormalities in the ER and mitochondria often affect both organelles under pathological conditions, resulting in altered production and transport of proteins, lipids, and other substances [8, 9] This can affect other organelles and change or destroy the physiological and biochemical activities and structure of cells [10, 11]. These abnormalities often have a significant effect on the occurrence and development of diseases.

This review focusses on the synergistic mechanisms between the ER and mitochondria, and their crosstalk with other organelles under physiological and pathological conditions.

### ER, the material synthesis center and mitochondria, the material oxidative metabolism center

The ER is considered the largest single dynamic membranous structure in eukaryotic cells, and has multiple different structural domains, including sheets, tubules and the nuclear envelope. Multiple functions, especially the synthesis, folding, and post-translational modification of proteins, lipid biogenesis and calcium (Ca^2+^) metabolism, can be flexibly realized through continuous structural reorganization [12]. It is identified as a key sensor and signal platform, and the main communication and exchange station between macromolecules and other organelles [6–9]. Therefore, the ER can be described as the cellular center of material synthesis.

Mitochondria are half-independent organelles with double functionally distinct and separate membranes, comprising the outer and inner mitochondrial membranes, which capsulize the matrix compartments and intermembrane space. The outer membrane is lipophilic and provides a channel for the import and export of small molecules. The inner membrane of mitochondria has a large number of invaginated folds, forming mitochondrial cristae, where the electron transport chain (ETC) are mainly located [13]. The mitochondrial oxidative phosphorylation system is the center of cellular metabolism and energy generation in eukaryotic cells. The ETC, which phosphorylates adenosine diphosphate into adenosine triphosphate (ATP), is also involved in other important biological processes, including cell death, reactive oxygen species (ROS) production, reproduction, inflammation, thermogenesis, and glucose and lipid metabolism [14, 15]. Mitochondria are also an important signal hub for lipid transport, Ca^2+^ signaling, ER stress, apoptosis, and autophagy [16]. It can change its structure and function in response to changing environmental conditions and plays a key role in a highly dynamic comprehensive network. Mitochondria form membrane contact sites with the ER, lipid droplets [17], Golgi apparatus, lysosomes, melanosomes, and peroxisomes [18]. These processes can conduct plasticity responses according to cellular conditions and subsequently interact with other organelles.

The regulation of metabolic pathways depends on the activities of different organelles to maintain energy homeostasis. The ER and mitochondria are the two main organelles that control cellular metabolism and energy production. Mitochondria generate energy on demand and are the end points of lipid, glucose, and glutamine catabolism and have a significant impact on the metabolic flux, energy charge, and cellular redox state. In contrast, the key step of glucose, lipid, and protein anabolism occurs in the ER, which enables a steady state metabolism in organelles [5]. Therefore, normal cellular metabolism is dependent on the activities of these two organelles, and their crosstalk.

### Crosstalk between ER and mitochondria

#### Communication platform between ER and mitochondria: ER-mitochondria (ER-MITO) contacts

Communication between the ER and mitochondria is essential for coordinating cellular responses. Abnormal communication between the ER and mitochondria is noted in various diseases, including obesity and diabetes, myocardial and cerebral ischemia, Alzheimer’s disease, Parkinson’s disease, Charcot Marie tooth disease, and cancer [19]. The physical link between them was discovered 40 years ago. Using electron microscopy, different sizes and shapes of tetras between the mitochondria and ER were found, which is our intuitive understanding of ER-MITO contacts [20]. The ER and mitochondria appear to interact inseparably through ER-MITO contacts in eukaryotic cells, but do not appear to fuse [21]. ER–MITO contacts appear stable because the two organelles stay in contact together even when they move along the cytoskeleton, and they still exist after cell division [22].

ER-MITO contacts may be a conserved feature of mitochondrial division. The ER seems to mark and participate in the division site because it maintains contact with the mitochondria during the entire mitochondrial fission process [23]. The ER wraps and constricts the mitochondria at the ER-MITO contacts. Mitochondrial fragmentation mediated by GTPase dynamin-related protein 1 (Drp1) mostly occurs at ER-MITO contacts [23, 24]. Newly formed mitochondrial DNA (mtDNA) remain in the daughter mitochondria after division. ER-MITO contacts may regulate mtDNA replication and coordinate the distribution of newly synthesized mtDNA between mitotic mitochondria [25]. However, further research is required to determine how mitochondrial fission is associated with mtDNA replication.

**Fig. 1:**
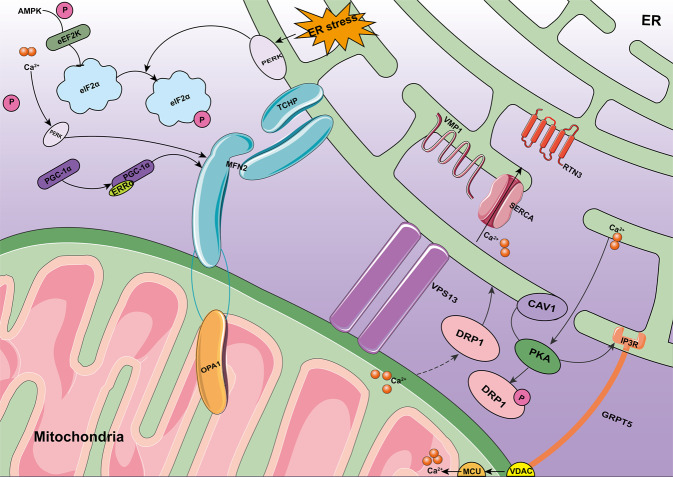
Crosstalk between the ER and mitochondria. ER-MITO contact provides a platform for crosstalk between these two organelles. Vps13 directly transfers lipids between the ER and mitochondria via hydrophobic channels spanning the entire N-terminal half of its rod. CAV1 acts as a PKA anchoring protein. ER stress and mTORC1 inhibition activate PKA. MTORC1 inhibition leads to PKA-mediated phosphorylation of DRP1 in an inhibitory manner. The IP3R-GRP75-VDAC1-MCU calcium regulatory axis mediates podocyte apoptosis by promoting mitochondrial Ca^2+^ overload. VMP1 regulates ER-MITO contact by activating the ER calcium channel, ATP2A/SERCA. TCHP regulates binding between mitochondria and the ER in an MFN2-dependent manner. TCHP expression causes mitochondria to break and relax their binding to the ER. AMPK is an upstream activator of PGC-1α that increases PGC-1α expression. eEF2K activity is regulated by AMPK phosphorylation at multiple sites. EEF2K can be inhibited by mTORC1-dependent phosphorylation of three different serine residues. AMPK activates eIF2α phosphorylation and prevents its translation.

ER-MITO contacts provide a platform for crosstalk between these two organelles (Fig. 1), which regulates various cellular activities, such as iron homeostasis, innate immune response, and metabolite exchange (Ca^2+^ and lipid) [26].

The ER-MITO contacts affects iron homeostasis. Iron is the basic element of nearly all organisms and participates in various biological processes as a key cofactor for many enzymes and proteins. Iron mainly exists in a reduced ferrous state in the intracellular environment. It is easy to dissolve and catalyzes the formation of ROS in cells [27, 28]. The cellular system can accurately adjust the iron content according to demand [29]. Disruption of the function of ER-MITO encounter structure (ERMES), which is the protein complex tethering ER-MITO contacts from the ER or mitochondria, on the ER or the mitochondrial surface induces iron accumulation [30]. The vacuolar protein sorting-associated protein 13 (Vps13)-D716H mutation inhibits the iron deficiency response of ERMES mutants [31, 32]. Additionally, mutations in the genes encoding Vps13 or Vps13A, B, and C are associated with neurodegenerative diseases in humans [33]. This provides a new direction for studying neurodegenerative diseases by analyzing the ER-MITO contacts and iron homeostasis.

Research based on the artificial immune system indicates that ER-MITO contacts play key roles in regulating the innate immune response. ER-MITO contacts can activate and mediate the signal transduction pathway downstream of pattern recognition receptors, and promote antiviral responses. The nucleotide-binding domain leucine-rich repeat (NLR) and pyrin domain containing receptor 3 (NLRP3) inflammasome comprises the receptor NLRP3 on the ER side and the adaptor apoptosis-associated speck-like protein containing a caspase recruitment domain on the mitochondrial side. NLRP3 induces caspase-1-dependent maturation of proinflammatory cytokines such as interleukin (IL)-1β and IL-18 [34, 35].

Researchers have identified a growing number of molecules that can regulate the number or tightness of ER-MITO contacts, including receptor expression enhancing protein 1 (REEP1) [36], caveolin-1 (CAV1) [37], protein kinase R-like endoplasmic reticulum kinase (PERK) [38], vacuole membrane protein 1 (VMP1) [39], and reticulon [40].

The regulation of ER-MITO contacts during ER stress has biological effects. REEP1 participates in ER amplification and is located in the ER. Mutated REEP1 is overexpressed in hereditary spastic paraplegia and loses its ability to promote interactions between the ER and mitochondria. Therefore, ER-MITO contacts are disrupted, which ultimately leads to neuroinflammatory degeneration of cortical neurons [36]. Silencing of CAV1 scaffold protein greatly reduces the contact sites between the ER and mitochondria, and increases cholesterol transport between organelles, whereas CAV1 expression has the opposite effect [37]. CAV1 reduces ER-MITO communication and inhibits ER-MITO contacts remodeling mediated by the cyclic adenosine monophosphate-dependent protein kinase (PKA)- Drp1 signaling axis [41]. The CAV1–PKA–Drp1 axis is a key regulator of organelle communication during ER stress. In conclusion, the dynamic relationship between the ER and mitochondria can fine-tune the amplitude and consequences of ER stress.

PERK is a novel molecular mediator of ER-MITO contacts sites and regulates inter-organelle crosstalk in ROS-induced cell death [38]. The ER activates the metabolism and energy response when glucose becomes the limiting condition for glucosamine synthesis and protein glycosylation. This process is mediated by PERK, which effectively recombines the ETC complex, but does not completely reconstruct the mitochondrial proteome [14, 15]. mtDNA encodes proteins that are structural components of the ETC and cannot function alone [42]. The ER-MITO contacts structure is not compact in PERK-deficient cells, and stress sensitivity mediated by ROS is abnormal. This abnormality leads to a reduction in death signals transmitted from the ER to the mitochondria, and the susceptibility to cell death is reduced [38]. PERK knockout restores mitochondrial Ca^2+^ levels and improves mitochondrial morphology in mitofusin 2 (Mfn2)-deficient cells [43]. This indicates an important tethering role of PERK in the maintenance of ER-MITO contacts.

VMP1 is an ER-localized metazoan-specific protein that plays a key regulatory role in autophagy. VMP1 deletion blocks autophagy and affects the morphology of mitochondria and ER-MITO contacts [39, 44]. There is a positive correlation between ER-MITO contacts and β-amyloids in the cerebrospinal fluid according to biopsy results of patients with Alzheimer’s disease. Nanomolar β-amyloid concentrations are sufficiently high to induce the expression of voltage-dependent anion channel 1 (VDAC1) and inositol 1,4,5 triphosphate receptor 3 (IP3R3), which increases ER-MITO and causes mitochondrial Ca^2+^ overload [45].

Reticulon 1a (RTN1a), RTN2b, and RTN3b increase ER-MITO contacts by 4.4 times, 2.5 times, and 1.3 times respectively, using engineered ascorbate peroxidase to map proteomes in live cells [40]. Therefore, RTN is considered an interesting new target for improving communication between the ER and the mitochondria.

### Role of mitochondria-associated membranes (MAMs) in the crosstalk between ER and mitochondria

ER and mitochondria form physical contact points through cholesterol-rich microdomains, called MAMs. The MAMs consist of a region of the ER that is reversibly connected to the mitochondria [46]. It has characteristics similar to those of ER and is rich in glucose-6-phosphate phosphatase and several lipid synthases. MAMs allow for a large amount of Ca^2+^ transfer from the ER to the mitochondria, thus stimulating mitochondrial bioenergetics or initiating apoptosis [47]. They are important hubs for glucose and insulin signaling, regulating liver metabolism, and adapting to nutritional cues [48].

MAMs are rich in Ca^2+^ transport channels, enzymes for lipid synthesis and transport, proteins encoded by oncogenes that regulate cellular signaling pathways, and tumor suppressors. Destruction of MAMs integrity activates various factors such as oxidative stress and apoptosis, leading to cellular damage [1, 19, 49]. Ca^2+^ instability depends on Ca^2+^ buffering capacity in mitochondria and MAMs integrity according to a study of striatal neurons derived from Huntington’s disease [50]. Mitofusin Mfn2 is highly enriched in MAMs [51]. Loss or silencing of Mfn2 increases the distance between the ER and the mitochondria, resulting in decreased Ca^2+^ flux at the MAMs [52]. Besides, MAMs are impaired in streptozotocin-induced diabetic mice and renal biopsy tissues of patients with diabetic nephropathy. The complete MAMs structure plays an important role in apoptosis in diabetic nephropathy [49]. Therefore, repairing the structure or function of MAMs may be a therapeutic approach for disease treatment.

### The role of Ca^2+^ in the crosstalk between ER and mitochondria

Intracellular Ca^2+^ transport is important for cell survival and ER and mitochondria are important Ca^2+^ storage organelles [53, 54]. Many binding molecules are regulated by Ca^2+^ and glucose in the membrane and lumen of ER and mitochondria [55]. Ca^2+^ pool homeostasis also promote the correct folding of some proteins in Ca^2+^-dependent molecules, such as glucose-regulated protein 78 and sarcoplasmic/endoplasmic reticulum Ca^2+^ ATPase [56]. Moderate mitochondrial Ca^2+^ loading has important physiological functions, such as cell signal transduction [57]. An increase in cytoplasmic Ca^2+^ concentration opens the permeability transition pore, which may accelerate mitochondrial cell death [58]. The ER maintains the cytoplasmic free Ca^2+^ concentration at a very low level.

Ca^2+^ in the mitochondria and ER flows between the two organelles [59]. Mitochondria and the ER dynamically adjust the Ca^2+^ concentration in the cytoplasm according to changes in the internal environment. Mitochondria in pancreatic β-cells release Ca^2+^, which affects the frequency and amplitude of the Ca^2+^ peak in the cytoplasm [60]. There are complex mechanisms of interaction between the ER and mitochondria in response to Ca^2+^ changes. Ca^2+^ enters the mitochondria from the ER through MAMs and plays an important role in mitochondrial division and apoptosis control [61]. Voltage-dependent anionic channels (VDAC) are an important class of channel proteins in the outer mitochondrial membrane. The metabolic flow and Ca^2+^ transmission between the ER and mitochondrial network requires VDAC [62, 63]. IP3R forms a complex with glucose-regulated protein 75 (GRP75) and VDAC, which allows crosstalk between signaling molecules and promotes Ca^2+^ transfer from the ER to the mitochondria [64]. Calreticulin provides buffering capacity in the ER and inhibits IP3R-mediated Ca^2+^ signaling through its high affinity and low capacity Ca^2+^-binding domain [65]. If the unfolded protein response cannot reduce cell stress, the cell increases ER-MITO contacts, Ca^2+^ release increases, and mitochondria assimilate Ca^2+^, which ultimately leads to apoptosis [1]. Ca^2+^ in the ER is rapidly released into the surrounding cytoplasm through IP3R. This exposes mitochondria to higher Ca^2+^ concentrations, which maintains ATP formation and prevents autophagy by reducing AMP-activated protein kinase (AMPK) activity [66, 67]. Blocking ER-mitochondrial Ca^2+^ transmission seriously damages mitochondrial ATP synthesis, increases the ratio of AMP/ATP, activates AMPK, and induces autophagy [68]. Similarly, stable knockout of mitochondrial Ca^2+^ uniporter inhibits mitochondrial Ca^2+^ uptake, reduces the oxygen consumption rate, activates AMPK, and induces autophagy [5, 69] (Fig. 1).

Abnormal Ca^2+^ transfer between ER and mitochondria is involved in many pathophysiological processes. In hepatocytes, obesity leads to an increase in Ca^2+^ transferred from the ER through MAMs, resulting in increased Ca^2+^ in the cytoplasm, whereas a high-Ca^2+^ environment is generally harmful to mitochondrial function. In contrast, downregulation of the IP3R1 Ca^2+^ channel and the ER-MITO tethering protein phosphofurin acidic cluster sorting protein 2 improves mitochondrial function, decreases cell stress, and improves glucose tolerance in obese mice [70]. Ca^2+^ flow between the ER and mitochondria influences the immune escape of cancer cells from mitochondria-mediated apoptosis [71]. Disturbance of the Ca^2+^ flux communication between the mitochondria and ER increases the MTOR-independent AMPK-dependent autophagic flux which cannot maintain anabolism required for cell homeostasis in cancer cells and induces cell death [72]. Therefore, regulation of Ca^2+^ crosstalk between the ER and mitochondria can be used in tumor therapy. Superparamagnetic iron oxide nanoparticles (SPIO-NPs) accumulate in the liver and destroy MAMs, which can directly change the Ca^2+^ steady state. This is because cyclooxygenase-2 (COX-2) overexpression enhances Ca^2+^ transfer from the ER to the mitochondria through MAMs to mediate SPIO-NP-induced apoptosis. SPIO-NPs promote COX-2 localization and enhance the physical crosstalk between COX-2 and the IP3R-GRP75-VDAC1 complex at MAMs. Celecoxib (a COX-2 inhibitor) reduces the destruction of MAMs in vivo and prevents liver injury [73]. Meanwhile, xestospongin B inhibits IP3R and induces cancer cell death [74]. The Bcl-2 pharmacological inhibitor ABT737 and cisplatin synergistically slow the progression of human ovarian cancer xenografts [75]. However, these methods require further research before they can be used clinically.

### The specific effect of ER and mitochondria crosstalk on lipid synthesis

Lipid synthesis requires the cooperation of multiple organelles [76, 77]. Phospholipids are the primary components of the cell membrane and are mainly synthesized in the ER and transported through vesicles [78–80]. MAMs control lipid membrane homeostasis in the ER and mitochondria and supports the transfer of different lipids. Large amounts of lipid are exchanged between the ER and the mitochondria [81]. Phosphatidylethanolamine is converted from phosphatidic acid in a series of steps: phosphatidylethanolamine enters the mitochondria through MAMs and finally returns to the ER. Phosphatidylserine synthase localizes to the ER face of MAMs and colocalizes with phosphatidylserine decarboxylase. Phosphatidylserine synthase and phosphatidylserine decarboxylase regulate the transport of phosphatidylserine from MAMs to mitochondria, which is the rate-limiting step of phosphatidylethanolamine synthesis in the mitochondria [82, 83].

Cholesterol and glycosphingolipids reportedly promote MAMs formation [84]. Sphingolipids, cholesterol, and proteins rapidly decompose and combine to form functional clusters in the cell membrane and play a role in membrane transport and cell signal transduction using efficient lipoprotein modules [85]. Glycosphingolipid-GM1 ganglioside accumulates on the ER membrane and promotes the juxtaposition of the ER and mitochondria in MAMs; this increases Ca^2+^ transfer between these organelles [86]. Therefore, some tumor cells inhibit mitochondrial metabolism and apoptosis signals by altering the lipid structure of the ER [1]. Most of the existing evidence regarding the role of different proteins in lipid transport is limited to cultured cells [87]. Further research should verify their functions in animal studies.

### Crosstalk between ER and other organelles

#### Crosstalk between ER and peroxisomes

**Fig. 2:**
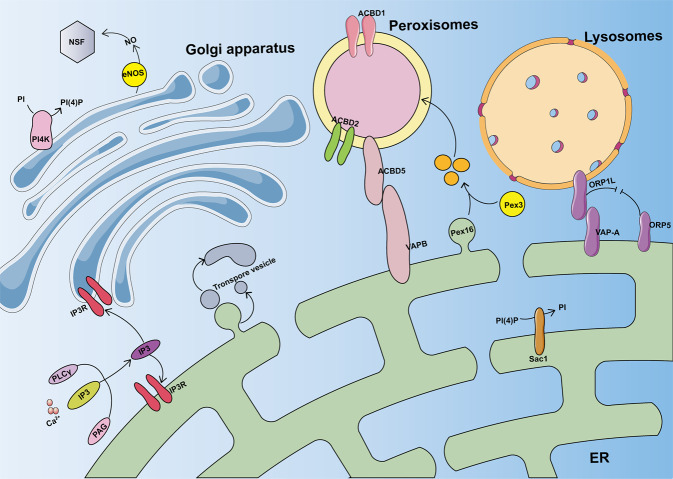
Crosstalk between the ER and other organelles. ORP1L contacts VAP-A to form ER lysosome contact sites under low cholesterol conditions. Pex16 containing vesicles derived from the ER, and Pex3 containing vesicles from the mitochondria can fuse and mature into peroxisomes. VAPA/B and ACBD5 constitute the ER-peroxidase contact site. ENOS closely associates with NSF and reduces the rate of protein transport from the Golgi to the plasma membrane. Ca^2+^ is released from intracellular stores through the binding of IP3 to IP3R.

The discovery of mitochondrial crosstalk with the ER implies that there may be contact sites with other organelles. Over 90% of mature peroxisomes in contact with the ER remained very close to the ER and limited their mobility [18, 88]. It is generally accepted that peroxisomes and ER play important roles in the biosynthesis of ether phospholipids. Ether phospholipid biosynthesis is initiated in the peroxisome and completed in the ER, which is required for the formation of glycosylphosphatidylinositol anchor proteins in the ER. Absence of 1-alkyl-2-acyl forms of glycosylphosphatidylinositol-anchored proteins may account for some of the complex phenotypes of peroxisomal disorders, such as psychomotor defects, mental retardation, and skeletal abnormalities [89]. Recent evidence has supported a new model of peroxisome biogenesis in which the new peroxisome hybridizes with the mitochondria and ER, and their derived characteristics are similar [90]. The ER peroxidase contact sites in human cells require ER-derived protein-associated proteins A and B and the tethered complex of peroxisome acyl-CoA-binding protein 5 [91]. Peroxisome biogenesis is derived from the mitochondria and ER. The vesicles containing peroxisomal biogenesis factor 16 (Pex16) and Pex3 come from the ER and mitochondria, respectively (Fig. 2). These two vesicles fuse and mature into peroxisomes [90]. Studying the crosstalk between the ER and peroxisomes is helpful in revealing the mechanism of peroxisome diseases.

### Crosstalk between the ER and Golgi apparatus

The structure of Golgi bodies is highly dynamic [92]. The Golgi apparatus transmits signals originating from the plasma membrane to other organelles to initiate intracellular signal transduction. Research in the past decade suggests that crosstalk between the ER and Golgi apparatus plays an important role in cell communication and in physiological and pathological responses [93].

Bidirectional lipid transport and protein secretion occur during juxtaposition of the ER and Golgi apparatus. The contact sites between the ER and Golgi membrane contain a PH domain, FFAT motif, and lipid transfer domain (ORD). The membranes are tethered by PH domains and the FFAT motif, allowing sterols to be transferred bidirectionally through the ORD [94]. ER receptors distinguish fully folded proteins from those that require further chaperone action; Golgi receptors combine misfolded goods and bring them back to the ER [95]. Endothelial nitric oxide synthase (eNOS) is a subtype of nitric oxide synthase that is mainly located in the Golgi apparatus. eNOS is closely related to N-ethylmaleimide-sensitive factor (NSF), which reduces the speed of protein transport from the Golgi to the plasma membrane [96] (Fig. 2). The bidirectional relationship between the Golgi complex and the endoplast/lysosome system and ER indicates that the precise localization of the Golgi complex is helpful in coordinating signal transduction between organelles during cell remodeling [97]. This helps maintain the stability of the intracellular environment.

### Crosstalk between the ER and lysosome

Lysosomal proteins are synthesized in the ER [98]. The distance between lysosomes and the ER at the contact sites is ~20 nm [99]. Lysosomal motility is controlled by small GTPases. Tethering and movement are controlled by a single molecular unit that is assembled on the late endosome/lysosomal GTPase Rab7 protein complex. Rab7 binds to GTP and forms a triple complex with Rab-interacting lysosomal protein and oxysterol-binding protein-related protein 1L (ORP1L) [100]. Under specific conditions, ORP1L is recognized by the ER transmembrane protein VAP-A through a targeting signal (phospho-FFAT) that allows the lysosome and ER to juxtapose [101] (Fig. 2). Lysosomes juxtaposed with the ER have multiple movements and may promote protein translocation to the ER membrane [102]. Lysosomes play an important role in expanding the structure and function of the ER.

### Crosstalk between mitochondria and other organelles

#### Crosstalk between mitochondria and the lysosome

Mitochondria and lysosomes interact in a variety of ways. Mitophagy, an autophagic process that specifically targets damaged mitochondria, is important in the crosstalk between the mitochondria and lysosomes [103, 104]. Dysfunctional mitochondria are isolated by autophagosomes which fuse with lysosomes, and the damaged mitochondria are degraded and recycled through the lysosomal chambers [105]. Mitophagy is activated to clear damaged mitochondrial proteins or partially damaged mitochondrial networks under extreme pressures, such as membrane potential loss and mitochondrial channel failure [106].

**Fig. 3:**
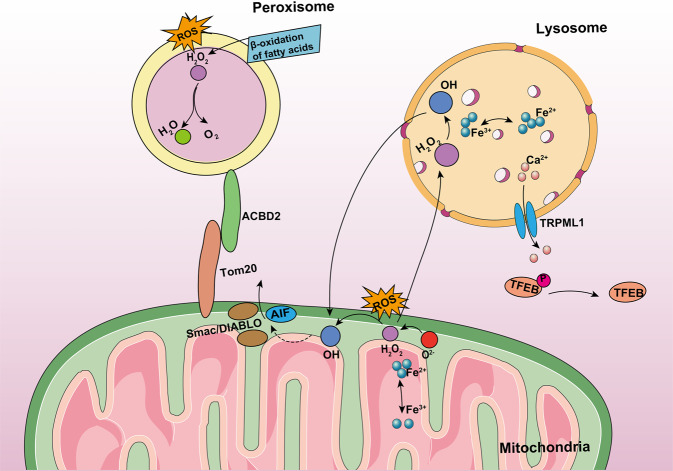
Crosstalk between mitochondria and other organelles. Elevated ROS levels within mitochondria causes TRPML1 to activate more lysosomal Ca^2+^ export channels to the cytoplasm. Elevated Ca^2+^ activates calcineurin, which dephosphorylates TFEB and increases cellular phagocytosis. H_2_O_2_ is one of the major members of ROS. H_2_O_2_ produced by mitochondria or other sites diffuses into lysosomes. Lysosome catalyzes the conversion of H_2_O_2_ to OH by the Fenton reaction. OH can disrupt the lysosomal membrane, resulting in leakage of lysosomal enzymes into the cytoplasm. Lysosomal enzymes can penetrate the outer membrane of mitochondria, allowing Smac/DIABLO and AIF to be released. Tom20 and ACBD2 participate in mitochondria–peroxisome connection, and promote substance transfer between the mitochondria and peroxisome.

Mitochondria and lysosomes in cardiomyocytes have common protein post-translational modifications that are complex and directed regulatory mechanisms [107]. Transcription factor EB (TFEB) is a mediator of lysosomal biogenesis and upregulation of TFEB expression can increase the number of lysosomes [108]. TRPML1 activates the lysosomal Ca^2+^ export channel to export Ca^2+^ to the cytoplasm in response to increase in mitochondrial ROS levels. Elevated Ca^2+^ levels activate calcineurin, dephosphorylate TFEB, and increase phagocytosis [109, 110] (Fig. 3). In addition, mitochondria generate mitochondria-derived vesicles that transport cargo to peroxisomes and lysosomes, and the cargo bound to lysosomes is finally degraded [111].

Hydrogen peroxide (H_2_O_2_) produced by mitochondria or other parts of the cell diffuses into lysosomes. Lysosomes catalyze the conversion of H_2_O_2_ to hydroxyl radicals via the Fenton reaction [112, 113]. OH can damage the lysosomal membrane and cause leakage of lysosomal enzymes and low-mass iron into the cytoplasm. Reactive iron may cause DNA damage by binding to mtDNA (or nuclear DNA) [112, 114, 115]. Lysosomal enzymes can penetrate the outer mitochondrial membrane. The release of cytochromes, the second mitochondria-derived activator of caspases/direct IAP binding protein with a low isoelectric point (Smac/DIABLO), and apoptosis-inducing factor (AIF) eventually induces cell death [115] (Fig. 3).

Defects in mitochondrial and lysosomal crosstalk may be associated with various human diseases, such as neurodegenerative diseases and cancer. The physiological functions of lysosomes and mitochondria are compromised in lysosomal storage disease, Parkinson’s disease, and mucolipidosis II and III [116]. Mitochondrial myopathies saturate lysosomal capacity, leading to lysosomal dysfunction and autophagosome accumulation [117, 118]. Destruction of the mitochondrial lysosomal axis and abnormal extracellular vesicles secretion lead to the aging process and many diseases [119].

Mito Blue is a recombinant fluorescent probe that initially acts on the mitochondria and then enters lysosomes. This probe can be used to explore the crosstalk between mitochondria and lysosomes in different cells [120]. Further exploration using other methods will facilitate research on the mitochondrial–lysosomal crosstalk.

### Crosstalk between mitochondria and peroxisomes

Peroxisomes are important organelles involved in ROS scavenging and maintenance of intracellular homeostasis. Peroxisomes and mitochondria are interrelated in function, which is termed as the “peroxisome–mitochondria connection” [121]. Peroxisome-mitochondria connections incorporate the metabolic cooperation of mitochondria and peroxisomes, such as the β-oxidation of fatty acids, phytanic acid α-oxidation, synthesis of bile acids and docosahexaenoic acids, glyoxylic acid metabolism, amino acid catabolism, polyamine oxidation, metabolism of ROS and nitrogen species [122]. Acyl-coenzyme A-binding domain 2 (ACBD2) is part of a mitochondria–peroxisome tethering complex that interacts with mitochondrial Tom20 in MA-10 cells; this interaction has important implications in steroid biosynthesis [121]. Peroxisomes are involved in mitochondrial lipid metabolism and respond to cellular or environmental disturbances by modifying their size, number, morphology, and function [123]. β-oxidation involves shortening of very long-chain fatty acids in peroxisomes, followed by transfer to the mitochondria for complete oxidation to prevent the toxic effects caused by their accumulation [121]. Therefore, abnormal peroxisomes can affect the physiological function of mitochondria through crosstalk, and interfere with lipid metabolism.

## Conclusions and future prospect

The synergistic interaction between ER and mitochondria and their crosstalk with other organelles play multiple key roles in material transportation, signal transmission, growth, and metabolism, which are related to disease pathogenesis. Abnormal crosstalk between them impede disorder of the internal environment and drive disease progression. A deeper understanding of organelle crosstalk can reveal more specific mechanisms of the occurrence and development of diseases. This would further facilitate the development of new approaches to treat diseases by regulating organelle homeostasis and normal organelle interactions.

However, the complete organelle interaction network remains unclear. During various diseases, abnormalities in some proteins and ions can affect multiple organelles. A prominent example is that abnormal changes in Ca^2+^ ions can interfere with interactions between organelles such as the ER, mitochondria, and lysosomes [55–57, 110]. Some other reported proteins playing a role in organelle crosstalk were summarized in Table 1. Hence, elucidating the complete map of the organelle crosstalk network should be a key focus of future biological research.

**Table 1:** Some reported proteins playing a role in organelle crosstalk.

Protein	Location	Function	References
VPS13	ER, mitochondria	Iron regulation	[33]
DRP1	ER, mitochondria	Control mitochondria division	[41]
NLRP3	ER	Modulate innate response	[34, 35]
CARD	Mitochondria	Modulate innate response	[34, 35]
REEP1	ER	Affect endoplasmic reticulum amplification	[36]
CAV1	ER, mitochondria	Cholesterol transport	[37]
PERK	ER, mitochondria	Cell death	[38, 43]
MFN2	ER, mitochondria	Affect mitochondrial morphology	[43, 51]
VMP1	ER	Autophagy	[39, 44]
VDAC	Mitochondria	The channel protein of Ca^2+^ transmission	[62]
IP3R	ER	The channel protein of Ca^2+^ transmission	[64, 65]
RTN	ER	Affect formation of the ER-MITO contact	[40]
AMPK	Mitochondria	Sensor of energy status	[72]
GSL	Lysosomal	Efficient lipoprotein modules	[84]
VAPA	Plasma membrane, intracellular vesicle	Affect vesicle transport, membrane fusion	[91]
ACBD5	Peroxisome	ER peroxidase contact site	[88, 91]
PEX	Peroxisome	Affect protein import into peroxisomes and peroxisome biogenesis	[90]
ORD	ER, Golgi apparatus	Part of the PH domain	[94]
NSF	Golgi apparatus	Reduce protein transport speed	[96]
ORP1L	Lysosome	Allow lysosome and ER to juxtapose	[100]
TFEB	Lysosome	Enable DNA-binding transcription factor activity	[108]

Furthermore, research on organelle crosstalk requires the establishment and development of new technologies and methods such as fault reconstruction, in vitro recombination, and fluorescent labeling. It is possible to describe the dynamic characteristics of organelle interactions with the help of real-time and quantitative tracing technology.

## Data Availability

All data that support the findings of this study are available from the corresponding author upon reasonable request.
